# Supplementary material for: The effect of physical activity on brain structure and cognitive function in the population-based cohort of LIFE-Adult Study
Source: eLife. 2026 Jul 15;15:RP109461. doi: 10.7554/eLife.109461 (PMC13372311; doi:10.7554/eLife.109461)
Supplement: Peer review history

# Editor report: eLife Assessment

**DOI:** 10.7554/eLife.109461.3.sa0

**Authors:** Andre F Marquand

**Affiliations:** Radboud University Nijmegen Netherlands

**Keywords:** Convincing, Valuable

This study presents a **valuable** examination of two measurements of physical activity (self-report and objective) in relation to widely studied structural MRI measures of the brain (hippocampal volume and BrainAGE) and cognitive function (Trail Making Test). Cross-sectional and longitudinal data were analyzed using established and validated methodology. The results **convincingly** suggest that brain health is more likely a cause of physical activity than an outcome of it, although limitations to the data could mask evidence of benefits to brain health but these are discussed by the authors. This work will be of interest to neurologists and epidemiologists studying the etiology of cognitive decline, to clinicians interested in advising patients on strategies for preserving brain health in aging, and to members of the lay public.

# Referee report: Reviewer #1 (Public review)

**DOI:** 10.7554/eLife.109461.3.sa1

Summary:

The authors investigated the relationship between physical activity (PA) and both structural (MRI) and cognitive brain health in the LIFE-Adult Study, with total baseline recruitment of 2576. Hippocampal volume, an MRI-derived BrainAGE marker, and scores from the Trail Making Test were used as outcomes, with the majority of participants measured at baseline and subsets also measured in a follow up session. The key findings were a lack of direct association between PA and outcomes, but longitudinal evidence for a higher BrainAge at baseline leading to lower physical capacity at follow-up. This supports a reverse-causation hypothesis in contrast to prevailing understanding of the positive effects of physical activity on brain health.

Strengths:

The Life-Adult study is a rich and carefully acquired dataset, with multiple follow-up time points. The statistical analyses were conducted carefully with appropriate control for confounds and multiple testing. The study design enables the important assessment for reverse causality. The authors are scrupulous in their consideration of a number of factors that could potentially bias their results, performing an age-stratified analysis, and emphasising discrepancies in PA measurements (specifically and age-reporting bias) across the dataset and other limitations.

Weaknesses:

This is an observational study with inconsistent measures of physical activity. Previous studies have used physical activity interventions, and might be more strongly weighted when considering evidence for these effects (specific confounders involved in interventions notwithstanding) .

The model identifying potential reverse causality is relatively limited - it seems possible/likely that brainAge could reflect more general health status, which would expand the potential range of factors underlying this observation. The authors comment on these possibilities.

The important quantitative actigraphy subset is small (n=227) as are the longitudinal subsets. Along with the discrepancy of physical activity/capacity at baseline and follow-up, and other complexities of the dataset, it is difficult to make firm conclusions. The authors point out that the actigraphy subset was quite inactive, and discuss this as a limitation.

# Referee report: Reviewer #2 (Public review)

**DOI:** 10.7554/eLife.109461.3.sa2

Summary:

This population-based cohort study found no evidence that physical activity, whether self-reported or objectively measured, positively influenced brain structure (hippocampal volume or BrainAGE) or cognitive function (Trail Making Test scores). Notably, longitudinal analyses suggested the opposite temporal relationship: a higher BrainAGE at baseline predicted higher physical capacity at follow-up, more in line with reverse causation rather than a neuroprotective effect of physical activity.

Strengths:

The study's statistical approach is thorough and well documented, and the inclusion of two measurements of physical activity (self-report questionnaire and objective accelerometer data) is a strength. The longitudinal aspect also represents a strength.

Weaknesses:

Several aspects of the measurement timing warrant consideration. Physical activity was assessed over 7-day periods, creating a potential mismatch with (commonly less dynamic) brain outcomes examined (hippocampal volume, BrainAGE), which may reflect cumulative exposures over longer timescales. Additionally, the asynchronous measurement protocol (cognitive testing preceding accelerometry, and the MRI occurring weeks after baseline visits) may introduce time lags that attenuate associations. The observed null associations may be influenced by timing misalignment rather than reflecting the absence of consistent effects of physical activity on brain health and cognition.

Other measurement characteristics also warrant consideration when interpreting the null findings. Physical activity was assessed using short-form self-report questionnaires and averaged accelerometer MET/day values, both of which have limited reliability. Additionally, the modest accelerometer subsample size and low/insufficient variation in activity levels observed in this cohort increase the likelihood of missing effects. These factors collectively raise the possibility that true physical activity-brain health associations may have been obscured.

The study's conclusions regarding brain health, structure, and cognitive functioning are broad despite the scope of the selection of outcomes examined. The analyses focus on hippocampal volume, BrainAGE (a global aging metric), and Trail Making Test performance (processing speed and executive function), while omitting other important neuroimaging markers such as cortical thickness, functional connectivity, or white matter microstructure. The null findings presented here cannot exclude positive effects of physical activity on broader constructs of brain health or cognitive functioning.

While the authors appropriately note the use of different physical activity instruments across time points (IPAQ at baseline, VSAQ at follow-up) in the limitations section, the discussion should more explicitly address the interpretive challenges this creates. The observed association between higher baseline brain age gap and lower follow-up physical activity may reflect: (1) a true temporal relationship, (2) an artifact of switching from behavior-focused (IPAQ) to capacity-focused (VSAQ) measurement, or (3) some combination of both. This ambiguity substantially limits causal inference.

Comments on the revised version:

I have briefly reviewed the responses to the reviewer comments, as well as the tracked changes in the expanded limitations section of the revised manuscript, and these adequately address my previous concerns.

# Author comment: Author response

**DOI:** 10.7554/eLife.109461.3.sa3

**Authors:** Polona Kalc, Robert Dahnke, Christian Sanders, Frauke Beyer, Andrea Zülke, Steffi Riedel-Heller, A Veronica Witte, Christian Gaser

**Affiliations:** Jena University Hospital Jena Germany; Jena University Hospital Jena Germany; University of Leipzig Medical Center Leipzig Germany; Bordeaux Population Health Leipzig Germany; Institute of Social Medicine, Occupational Health and Public Health, University of Leipzig Leipzig Germany; Institute of Social Medicine, Occupational Health and Public Health, University of Leipzig, Faculty of Medicine Leipzig Germany; Max-Planck-Institute for Human Cognitive and Brain Sciences Leipzig Germany; University of Jena Jena Germany

The following is the authors’ response to the original reviews.

**Public Reviews:**

**Reviewer #1 (Public review):**

Summary:

The authors investigated the relationship between physical activity (PA) and both structural (MRI) and cognitive brain health in the LIFE-Adult Study, with total baseline recruitment of 2576. Hippocampal volume, an MRI-derived BrainAGE marker, and scores from the Trail Making Test were used as outcomes, with the majority of participants measured at baseline and subsets also measured in a follow-up session. The key findings were a lack of direct association between PA and outcomes, but longitudinal evidence for a higher BrainAge at baseline leading to lower physical capacity at follow-up. This supports a reverse-causation hypothesis in contrast to the prevailing understanding of the positive effects of physical activity on brain health.

Strengths:

The Life-Adult study is a rich and carefully acquired dataset, with multiple follow-up time points. The statistical analyses were conducted carefully with appropriate control for confounds and multiple testing. The study design enables an important assessment for reverse causality. The authors are scrupulous in their consideration of a number of factors that could potentially bias their results, performing an age-stratified analysis, and emphasising discrepancies in PA measurements (specifically, age-reporting bias) across the dataset and other limitations.

Weaknesses:

This is an observational study with inconsistent measures of physical activity. Previous studies have used physical activity interventions, and might be more strongly weighted when considering evidence for these effects (specific confounders involved in interventions notwithstanding).

The model identifying potential reverse causality is relatively limited - it seems possible/likely that brainAge could reflect more general health status, which would expand the potential range of factors underlying this observation.

The important quantitative actigraphy subset is small (n=227), as are the longitudinal subsets. Along with the discrepancy of physical activity/capacity at baseline and follow-up, and other complexities of the dataset, it is difficult to make firm conclusions. The authors point out that the actigraphy subset was quite inactive.

We would like to thank the reviewer for their valuable feedback. We agree with the limitations mentioned, and we have extended the discussion section in order to address the drawbacks more effectively. In particular, we agree that the null findings of this study do not suggest that physical activity has no effect on the brain; for such a conclusion, an intervention study would be necessary.

Furthermore, we agree that BrainAGE might reflect a more general health status. Although we excluded images of individuals with visible acquired brain injuries, we did not control for other medical conditions (e.g. hypertension or diabetes), which may have affected the results.

Please see the revised discussion parts in the response below.

**Reviewer #2 (Public review):**

Summary:

This population-based cohort study found no evidence that physical activity, whether self-reported or objectively measured, positively influenced brain structure (hippocampal volume or BrainAGE) or cognitive function (Trail Making Test scores). Notably, longitudinal analyses suggested the opposite temporal relationship: a higher BrainAGE at baseline predicted higher physical capacity at follow-up, more in line with reverse causation rather than a neuroprotective effect of physical activity.

Strengths:

The study's statistical approach is thorough and well-documented, and the inclusion of two measurements of physical activity (self-report questionnaire and objective accelerometer data) is a strength. The longitudinal aspect also represents a strength.

Weaknesses:

Several aspects of the measurement timing warrant consideration. Physical activity was assessed over 7-day periods, creating a potential mismatch with (commonly less dynamic) brain outcomes examined (hippocampal volume, BrainAGE), which may reflect cumulative exposures over longer timescales. Additionally, the asynchronous measurement protocol (cognitive testing preceding accelerometry, and the MRI occurring weeks after baseline visits) may introduce time lags that attenuate associations. The observed null associations may be influenced by timing misalignment rather than reflecting the absence of consistent effects of physical activity on brain health and cognition.

Other measurement characteristics also warrant consideration when interpreting the null findings. Physical activity was assessed using short-form self-report questionnaires and averaged accelerometer MET/day values, both of which have limited reliability. Additionally, the modest accelerometer subsample size and low/insufficient variation in activity levels observed in this cohort increase the likelihood of missing effects. These factors collectively raise the possibility that true physical activity-brain health associations may have been obscured.

The study's conclusions regarding brain health, structure, and cognitive functioning are broad despite the scope of the selection of outcomes examined. The analyses focus on hippocampal volume, BrainAGE (a global aging metric), and Trail Making Test performance (processing speed and executive function), while omitting other important neuroimaging markers such as cortical thickness, functional connectivity, or white matter microstructure. The null findings presented here cannot exclude positive effects of physical activity on broader constructs of brain health or cognitive functioning.

While the authors appropriately note the use of different physical activity instruments across time points (IPAQ at baseline, VSAQ at follow-up) in the limitations section, the discussion should more explicitly address the interpretive challenges this creates. The observed association between higher baseline brain age gap and lower follow-up physical activity may reflect: (1) a true temporal relationship, (2) an artifact of switching from behavior-focused (IPAQ) to capacity-focused (VSAQ) measurement, or (3) some combination of both. This ambiguity substantially limits causal inference.

Thank you for a thorough review of the manuscript. We appreciate the opportunity to consider the limitations in more detail. As you highlight, the null findings could be caused by a variety of reasons and changes may have occurred in white matter microstructure or functional connectivity that could not be observed using our chosen measures. We have expanded the discussion to address these and other issues (p. 12):

“However, our results should be interpreted with caution, due to the limited sample size, potential attenuation of effects resulting from measurement error in the assessment of physical activity/capacity and the shift from an activity-based measure at baseline to a capacity-based measure at follow-up. This change limits the interpretability of longitudinal effects, as observed associations may reflect both changes in the underlying construct being measured and true changes in the relationship over time.

Strengths and Limitations

The results of this cross-sectional observational study may be affected by various factors, including bias in self-reported physical activity [48, 49], accelerometer measurement error [59, 60] and reverse causality, among others. Moreover, our results may also be affected by the general medical status of the participants, since we did not control for other diseases within the sample. In fact, BrainAGE may reflect overall health and the cumulative impact of various factors (including previous physical activity) on brain health over an extended period of time. Furthermore, our analysis focused on only a few cognitive and structural brain measures. While we did not observe any changes in hippocampal volume or BrainAGE, this does not exclude the possibility of changes in white matter integrity or functional connectivity. Another limitation of this observational study was the time lag between physical activity measurements and MRI scanning, which may have reduced the observed effects. Although the longitudinal design is a major strength of this study, attrition of participants at follow-up may have affected our estimates. Furthermore, the use of cross-lagged panel model design in the longitudinal setting has frequently been criticised for not distinguishing between within-person changes and between-person differences [61, 62], and our adapted design suffers from these limitations, as well as others arising from the use of different instruments to measure the construct related to physical activity at each time point (IPAQ and VSAQ). Nevertheless, compared to large volunteer-based cohorts such as the UK Biobank, the registry-based recruitment strategy of the LIFE-Adult Study may be less susceptible to healthy volunteer bias, although we cannot entirely eliminate the possibility of volunteer bias among the participants with accelerometry data in our case.

Direct comparisons are limited in the absence of harmonised recruitment and assessment protocols.”

Additionally, please note that in the Summary sentence ‘Notably, longitudinal analyses suggested the opposite temporal relationship: a higher BrainAGE at baseline predicted higher physical capacity at follow-up, more in line with reverse causation rather than a neuroprotective effect of physical activity’

The opposite is actually true; higher BrainAGE at baseline predicted lower physical capacity at follow-up.

**Recommendations for the authors:**

**Reviewer #1 (Recommendations for the authors):**

The analysis and discussion are somewhat limited. More detail and discussion of the demographic features of the study dataset, and perhaps a stronger concluding position regarding the potential impacts of PA on brain and cognition would be helpful - this might also be integrated into the abstract.

Thank you for pointing this out. As both of the reviewers have highlighted that the discussion is limited, we appreciate the opportunity to revise it (see also the response above). We hope that it now gives a more comprehensive interpretation of the results.

We have also updated our abstract to include a bit stronger concluding position:

“Physical activity is believed to positively influence brain health and cognition and is considered a modifiable lifestyle factor that may protect against cognitive decline and neurodegeneration. In this observational study, we investigated the cross-sectional and longitudinal effects of self-reported total and moderate-to-vigorous physical activity on cognitive scores on the Trail Making Test (TMT-A and TMT-B), hippocampal volume, and BrainAGE, in a large population-based cohort from the LIFE-Adult Study (n = 2576). Furthermore, we examined the effect of objectively measured physical activity on brain structure in a subgroup with available accelerometry data (n = 227). Multiple linear regression analyses did not show any positive effects of self-reported or objectively measured physical activity on hippocampal volume or processing speed and executive function. Longitudinal path analyses suggested a potential for reverse causation, where a higher BrainAGE at baseline was associated with lower physical capacity at follow-up. Additionally, we observed an age-related bias in the self-reporting of physical activity, indicating that older individuals tend to overestimate their level of activity. Future interventions targeting middle-aged adults may be necessary to raise awareness of potential misperception and encourage increased physical activity.”

There might be more careful inspection of alternative models and dissection of the impact of covariates (e.g. smoking, which is very prevalent in this cohort). For example, did PA show any benefit specifically in the "non-smoker" vs. "smoker" subgroups?

Thank you for this suggestion. We decided against including analysis of various subgroups, as this would have shifted the focus of the manuscript. However, we do provide the results of the analysis with the interaction term here. There was no evidence that smoking status moderated the association between self-reported physical activity and BrainAGE at follow-up (*p* = .192). In both non-smokers and smokers, physical activity was not significantly associated with BrainAGE at follow-up (b = 0.038, SE = 0.033, *p* = .242 and b = −0.028, SE = 0.038, *p* = .471, respectively).

The age-dependent reporting bias seems important and should be assessed and discussed in more detail - it could have important implications for other studies. Why might this occur?

We appreciate your drawing more attention to this point. As you have mentioned, it can have important implications for other studies, suggesting that objective measures of activity should, if possible, be used alongside self-report questionnaires. We have expanded on this topic in more detail in the revised discussion (p. 11):

“This age-dependent reporting bias was previously demonstrated by other studies, where higher age was associated with overreporting activity levels [51-54]. Overreporting could stem from worsening recall, socially desirable responses, and the subjective nature of self-report questionnaires, which also depend on a person’s physical fitness [51]. Future (observational) studies would greatly benefit from including both accelerometer and self-reported measures of physical activity.”

The reverse causation result could also be discussed (and possibly analysed) in more detail - what might the neurobiological mechanisms underlying this be? Is general health a factor - were confounds like smoking/health assessed here?

Thank you for raising this important point. We have not added confounds other than age here, as we did not have enough degrees of freedom to add additional parameters to the model. However, we agree with you that general health might have played an important role here, although we don’t have a specific measure for it. We have expanded upon these limitations in the revised discussion (p. 12):

“Similarly to Hofman et al. [62] and Rodriguez-Ayllon et al. [63], who found a bidirectional association between physical activity and brain structure, with a more consistent pattern of brain structural measures affecting physical activity, the results of our path analysis partially supported the reverse causality explanation, indicating that baseline brain health influences follow-up physical capacity, rather than baseline physical activity affecting follow-up brain health. Possible mechanisms may involve decreasing health status with age-related mitochondrial dysfunction [64, 65] and potential low-grade inflammation, which could result in fatigue [66] and a possible decline in fitness and physical capacity. Further studies are necessary to investigate this in more detail.”

Results should contain greater detail; in particular, they should summarise key results from tables and not rely on the reader to carefully look through all figures. Reporting relatively non-informative results (e.g. entirely unadjusted model results) does not add much.

We appreciate your feedback. We have tried to summarise the results in greater detail, reporting statistical values within the text (e.g., main results, p. 7):

“The results indicate no statistically significant effects of self-reported total PA on brain structure (β = -0.029, *p* = .137 and β = 0.035, *p* = .137 for hippocampal volume and BrainAGE, respectively). There is a statistically significant effect of total self-reported PA on cognitive function, indicating that higher levels of PA lead to higher time scores on TMT-B (β = 0.053, *p* = .042). Similar results can be observed for MVPA, however, the results do not survive the correction for multiple comparisons (β = 0.045, *p* = .072). The analysis of objectively measured PA indicated no statistically significant effects on brain structure (β = -0.036, *p* = .582 and β = -0.076, *p* = .393 for hippocampal volume and BrainAGE, respectively).”

We report the results of the unadjusted model to provide greater transparency, in line with the Strengthening the Reporting of Observational Studies in Epidemiology (STROBE) guidelines.

**Reviewer #2 (Recommendations for the authors):**

Please review the manuscript for consistent use of abbreviations and definitions (e.g., write out BDNF line 49). Specifically, definitions and language to describe BrainAGE, brain age, brain age gap, neuroimaging-derived biomarker of brain ageing, Brain Age Gap Estimate, BA, BrainAGE (BA), etc. would benefit from consistency.

Thank you very much for this observation. We have revised the manuscript in the hope that it is now more consistent. Specifically, we spelled out the ‘brain-derived neurotrophic factor’ and replaced the abbreviation 'BA' in Figure 2 with BrainAGE. However, we acknowledge that there are already many naming inconsistencies within the field. We therefore tried to follow the different authors' notations, which were established within the field: we used 'brain age gap' when referring to James Cole's model (or the concept in general), and 'BrainAGE' for our own.

We thank both reviewers for their helpful feedback, which has improved our manuscript.
